# Implant design affects walking and stair navigation after total knee arthroplasty: a double-blinded randomised controlled trial

**DOI:** 10.1186/s13018-021-02311-x

**Published:** 2021-03-06

**Authors:** Dimitrios-Sokratis Komaris, Cheral Govind, Andrew James Murphy, Jon Clarke, Alistair Ewen, Hollie Leonard, Philip Riches

**Affiliations:** 1 Tyndall National Institute, University College Cork, Lee Maltings Complex Dyke Parade, Cork, T12 R5CP Ireland; 2 Department of Biomedical Engineering, University of Strathclyde, Glasgow, Scotland; 3 School of Medicine, University of Dundee, Dundee, Scotland; 4 Orthopaedic Department, Golden Jubilee National Hospital, Clydebank, Scotland

**Keywords:** Knee prosthesis, Fixed bearing, Mobile bearing, Implant congruency, Range of motion, Motion analysis

## Abstract

**Background:**

Dissimilar total knee arthroplasty implant designs offer different functional characteristics. This is the first work in the literature to fully assess the Columbus ultra-congruent mobile (UCR) system with a rotating platform.

**Methods:**

This is a double-blinded randomised controlled trial, comparing the functional performance of the low congruent fixed (CR DD), ultra-congruent fixed (UC) and UCR Columbus Total Knee Systems. The pre-operative and post-operative functional performance of twenty-four osteoarthritic patients was evaluated against nine control participants when carrying out everyday tasks. Spatiotemporal, kinematic and kinetic gait parameters in walking and stair navigation were extracted by means of motion capture.

**Results:**

The UC implant provided better post-operative function, closely followed by the UCR design. However, both the UC and UCR groups exhibited restricted post-operative sagittal RoM (walking, 52.1 ± 4.4° and 53.2 ± 6.6°, respectively), whilst patients receiving a UCR implant did not show an improvement in their tibiofemoral axial rotation despite the bearing’s mobile design (walking, CR DD 13.2 ± 4.6°, UC 15.3 ± 6.7°, UCR 13.5 ± 5.4°). Patients with a CR DD fixed bearing showed a statistically significant post-operative improvement in their sagittal RoM when walking (56.8 ± 4.6°).

**Conclusion:**

It was concluded that both ultra-congruent designs in this study, the UC and UCR bearings, showed comparable functional performance and improvement after TKA surgery. The CR DD group showed the most prominent improvement in the sagittal RoM during walking.

**Trial registration:**

The study is registered under the clinical trial registration number: NCT02422251. Registered on April 21, 2015.

**Supplementary Information:**

The online version contains supplementary material available at 10.1186/s13018-021-02311-x.

## Background

Advancements in the design of total knee arthroplasty (TKA) prostheses have led to the commercialisation of more than 150 types of knee implants [1] with potentially different functions. For example, in fixed-bearing designs, the polyethylene sheet is fixed upon the underlying tibial component, whilst in mobile bearings the insert can rotate short distances inside the metal tibial tray; bearing congruency also changes the level of conformity between the femoral section and the bearing surface, which in theory affects mobility, contact forces, and polyethylene wear. The possible advantages of mobile over fixed bearings (e.g., [2]), posterior cruciate ligament (PCL) retention versus substitution (e.g., [3]), and the clinical outcomes of different bearing congruencies (e.g., [4]), component fixation methods (e.g., [5]) and patellar resurfacing techniques (e.g., [6]) have all been extensively investigated. In the present study, three types of Columbus® total knee prostheses (B. Braun Aesculap, Tuttlingen, Germany) with different platform designs, degrees of congruency and PCL management are compared.

Variations of the Columbus knee have been previously investigated, either between different bearing designs or in comparison to other commercially available implants with no statistical differences found between mobile and fixed bearings, and with similar Oxford Knee Scores and passive sagittal range of motion (RoM) between all investigated commercially accessible prostheses (Table 1). Additionally, studies investigating other commercially available knee implants have had similar findings. For example, Urwin et al. [18] reported no significant differences between fixed (Sigma® Fixed Bearing Knee System, De Puy International) and mobile bearings (Sigma® Rotating Platform Knee System, DePuy International) in spatiotemporal, kinematic and kinetic measurements (stride length and time, gait velocity, flexion angles during walking, knee RoM and maximum knee adduction moment) at 9 months post-operatively. Additionally, TKA patients in the same study walked with greater minimum knee flexion and reduced knee adduction moment when compared to the control group.

**Table 1:** Summary of papers with Columbus knee implants

Study	Prostheses	Type of bearing	No of knees	Knee passive flexion RoM (°)		Significant differences found
				Pre-operative	Post-operative	
Lampe et al. [7]	Columbus CR	Fixed	52	111 ± 15	113 ± 13	No^a^
	Columbus RP	Mobile	48	109 ± 12	115 ± 11	
Goebel and Schultz [8]	Columbus Knee	Fixed	109	-	-	Yes^c^
	NexGen Full Flex	Fixed	22	-	-	
Jung et al. [9]	Columbus PS	Fixed	197	128.4 ± 16.2	131.8 ± 10.7	No^c^
	Scorpio PS	Fixed	187	124.5 ± 19.9	130.2 ± 14.2	
Hakki et al. [10]	Columbus DD/UC	Fixed	79	94.7	110.4	-
Marques, Daniel [11]	Columbus CR	Fixed	45	110.6 ± 15.5	114.3 ± 9.3	No^a^
	Columbus RP	Mobile	42	109.4 ± 12.7	117.7 ± 10.9	
Luzo et al. [12]	Columbus PS	Fixed	196	-	-	Yes^b^
Kim et al. [13]	Columbus UC	Fixed	73	115.5 ± 10.3	125.6 ± 9.1	No^a^
	E-motion UC	Mobile	73	114.1 ± 9.6	123.7 ± 9.7	
Lutzner et al. [14]	Columbus UC	Fixed	63	102.9 ± 14.6	112.2 ± 11.8	No^c^
	Columbus PS	Fixed	64	102.1 ± 13.1	115.1 ± 13.0	
Yoon and Yang [15]	Columbus UC	Fixed	233	125.0 ± 13	-	-
Yoon and Yang [16]	Columbus UC	Fixed	105	125 ± 13	127 ± 8	No^a^
	E-motion FP	Mobile	95	129 ± 10	120 ± 5	
Fuchs et al. [17]	Columbus CRDD	Fixed	187	106.3 ± 20.2	114.0 ± 12.1	Yes^b^

*CR* cruciate retaining, *DD* deep dish, *RP* rotating platform, *PS* posterior stabilised, *UC* ultra-congruent, *FP* floating platform

^a^Between fixed and mobile bearings

^b^Between pre-operative and post-operative assessment

^c^Between different commercially available knee designs

However, commonly used clinical scores, such as knee RoM and patient-reported outcome measures, are insensitive and can be inadequate due to the presence of floor and ceiling effects [19]. Quantitative biomechanical analyses of TKA patients during activities of daily living, including more functionally challenging activities such as stair ascent and descent, may be more appropriate and statistically powered to evidence differences between prostheses. To further explore whether implant design paradigm variations result in significant differences, this study evaluates the full biomechanical performance of three different bearing configurations of Columbus knee replacement implants and the age-matched natural knee; the hypothesis being that the mobile high congruent bearing will facilitate more natural movements during everyday tasks.

## Material and methods

### Recruitment

Following appropriate ethical approvals, volunteers were recruited to this randomised, controlled, double-blinded study (ClinicalTrails.gov identifier: NCT02422251). Volunteers were sought from patients scheduled for unilateral TKA at the Golden Jubilee National Hospital, Clydebank, Scotland, between August 2015 and June 2017. Patients were excluded if they had had a hip or knee replacement procedure in the previous 12 months, had previous ankle surgery or past neurologic history (e.g., stroke). Recruits gave written informed consent and were blindly randomised using sequentially numbered opaque sealed envelopes, to receive one of three designs: a low congruent fixed (CR DD, cruciate-retaining deep-dish), a high congruent fixed (UC, ultra-congruent) or a high congruent mobile (UCR, ultra-congruent rotating platform) knee bearing (Columbus Total Knee Systems, B. Braun, Melsungen, Germany). A nominated person, independent of the approach and consent of the patient, opened the envelopes and informed the hospital team of the randomisation. Both the patient and the research team responsible for the motion capture and data analyses were blinded to the implant allocation.

The high congruent bearings used in the study are posterior stabilised and require the posterior cruciate ligament (PCL) to be resected; the CR DD on the other hand has a cruciate retaining design. All tibial and femoral implant components were fixed with cement, and surgeries were performed using the OrthoPilot navigation system. If, intra-operatively, the allocated bearing was deemed inappropriate for a given patient, the operating surgeon could choose a more suitable implant, and the patient was excluded from the study. Operations were performed by three consultant orthopaedic surgeons who all used the three study implants in their routine clinical practice. Age-matched, asymptomatic, control volunteers were recruited from community groups and social clubs. Control participants were excluded if they had a previous lower limb joint replacement procedure, ankle surgery, or any musculoskeletal, neurological, or sensory deficit.

**Fig. 1:**
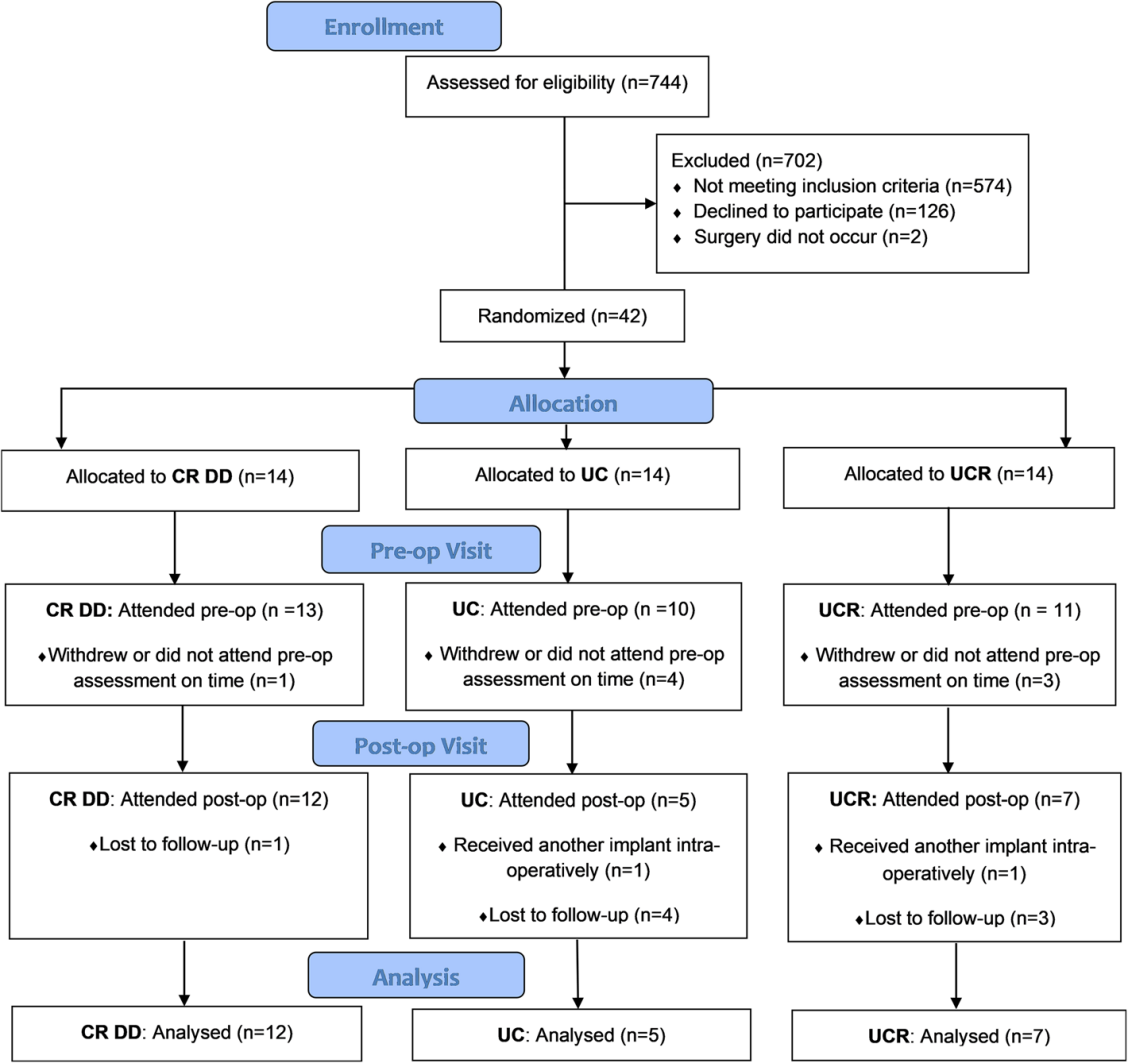
CONSORT enrollment flow diagram

Seven hundred and forty-four patients were assessed for eligibility, of which forty-two patients were recruited in the study (Fig. 1), along with nine control participants. Patients’ biomechanical performance was assessed pre-operatively and 1 year post-operatively in levels walking, stair ascent, and stair descent. Control participants attended a single motion capture session only. Out of forty-two, eight patient participants failed to attend their pre-operative assessment at the designated time period of 1 week before their operation and were excluded from the study; another eight patients withdrew post-operatively for health reasons; finally, two patients did not receive a Columbus implant upon surgery. Therefore, a total of twenty-four patients were included in this analysis, of which, twelve, five, and seven received the CR DD, UC, and UCR implants, respectively.

### Demographics

Gender, age, and body mass index (BMI) were recorded for all groups, and affected knee side was noted for the patient groups (Table 2). Pre- and post-operative Oxford Knee Scores (OKS) were also logged by clinicians during hospital visits. A patient’s OKS is generated from a twelve-item questionnaire, assessing the difficulty and pain levels during common activities of daily living; the best possible outcome is 12, and the score giving the worst is 60.

**Table 2:** Participant demographics

Demographics	CR DD	UC	UCR	Control
*n*	12	5	7	9
Male/female	11/1	1/4	3/4	3/6
Age, mean ± std (years)	66.6 ± 4.0	68.4 ± 8.8	70.1 ± 6.2	70 ± 6.4
Affected side (L/R)	5/7	4/1	5/2	-
Pre-op BMI, mean ± std (kg/m^2^)	30.0 ± 2.5*	32.0 ± 5.9*	30.3 ± 3.3*	24.0 ± 3
Post-op BMI, mean ± std (kg/m^2^)	30.1 ± 2.5*	31.6 ± 6.1*	29.6 ± 3.4*	-
Pre-op OKS, mean ± std	35 ± 7	42 ± 4	36 ± 5	-
Post-op OKS, mean ± std	**24 ± 8**^**†**^	**19 ± 4**^**†**^	**22 ± 8**^**†**^	-

^*, †^ Statistically significant difference between implant and control groups, and between pre-op and post-op visits, respectively (*P* <.01)

Age, BMI, and OKS between groups were compared using one-way ANOVA tests with a Bonferroni correction. Patients showed significantly higher BMI values than the control group (*P*<0.01). No statistically significant differences in age and OKS were noted between all groups. *T*-tests showed no change in BMI post-operatively; OKS was significantly improved after surgery for all implant groups (*P*<0.01). Patient satisfaction was also assessed 1-year after TKA using a five-point Likert scale (1, very dissatisfied; 2, dissatisfied; 3, unsure; 4, satisfied; 5, very satisfied), with twenty-two and two patients reporting to be very satisfied and satisfied, respectively.

### Instrumentation

Motion capture was carried out with twelve Vicon (Oxford Metrics, Oxford, UK) T-series cameras and four Kistler (Winterthur, CH) force platforms, sampling at 100 and 1000 Hz, respectively. Prior to each recording, a calibration weight of 200 N was placed on top of each force plate, and the error in the recorded value was logged. Following the calibration of the motion capture system, the highest camera error (mm) was also noted. Both errors were low and consistent across all recordings (force plates 1.9 ± 0.9%, cameras 0.19 ± 0.009 mm).

Male participants wore Lycra shorts and comfortable footwear; female participants additionally wore Lycra t-shirts. Following anthropometric measures, thirty-five 14-mm retroreflective markers were fixed to anatomical body locations as per the full-body Plug-in Gait biomechanical model. A knee alignment device (KAD) was used during the static calibration of the participants.

### Recordings

Initially, participants walked for 10 m at a comfortable walking speed. No other instructions or information about the existence of force platforms was given. Both starting and finishing positions were clearly marked on the floor with a coloured tape. A minimum of six walking trials were captured per participant, with extra being recorded if the number of clean force plate strikes was lower than five.

**Fig. 2:**
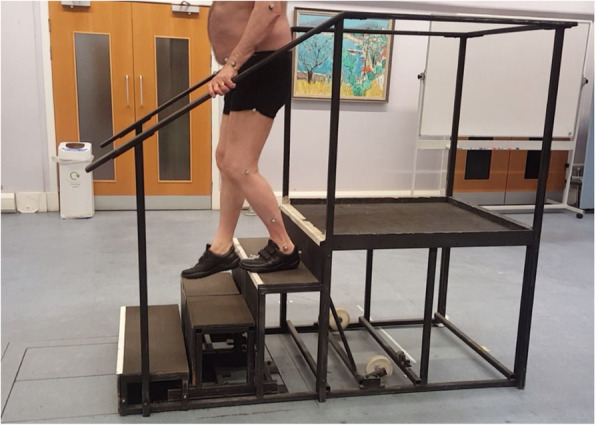
Staircase with a step height and length of 185 mm and 280 mm, respectively

For the stair ascent and descent tasks, a four-step staircase with two handrails was fixed adjacent to two force platforms (Fig. 2). The steps’ rise and going were 185 mm and 280 mm, respectively. The second step of the staircase was composed of two parts that were individually mounted on different force platforms. To ensure that no noise was captured by the force transducers during the gait’s double support phase, 1-cm gaps were left between the second and its two neighbouring steps. The surfaces of the force plates under the second step were also raised by 370 mm in the acquisition software, to compensate for the further displacement from the piezoelectric sensors. Participants were instructed to initiate the trial at a two-step distance from the construction and climb the staircase with their own preferred manner. The use of handrails was not restricted, and no instructions were given as to which foot ought to initiate ascent or descent. Upon reaching the plateau at the top of the staircase, participants were asked to turn around and descend back to the start position. A minimum of five ascent and five descent trials per participant was recorded.

### Processing

Trials were cropped to discard frames with low marker visibility using the Vicon Nexus software. At least one gait cycle per limb per trial was considered. Gaps of less than five frames in the markers’ trajectories were filled with Woltring quintic spline fills, whereas larger gaps were filled with rigid body or pattern fills. Marker and force recordings were filtered using a 4th order Butterworth filter with cutoff frequencies of 6 and 300 Hz, respectively. Gait events were automatically detected from force and marker data, and all trial data were normalised to 100% of the gait cycle using custom MATLAB scripts. Finally, all gait cycles for each lower limb were averaged to a single data set per person and type of activity.

Differences in gait parameters between patient groups, both pre-operatively and post-operatively, and the control group were compared. Functional metrics were categorised into spatiotemporal (walking speed, cadence, stride time, contralateral foot-off and heel-strike instances, and duration of double support), kinematic (peak knee joint movements, knee RoM and knee flexion at heel-strike and toe-off) and kinetic parameters (peak knee external moments and power). All kinematic measurements of the patient and control participants are with reference to the operated and both limbs, respectively.

Since participants ascended and descended the staircase in their own manner, the adopted movement strategies potentially affected the biomechanical functioning. Whilst the use of handrails could expect to reduce the weight bearing of the pathological leg and affect knee joint moments, knee kinematics have been found to be unaffected in old adults [20]. Therefore, stair navigation performance was assessed with the use of spatiotemporal and kinematic parameters only, whilst related kinetic metrics are reported only as supplementary data. Secondly, given that a step-by-step strategy could influence all concerned metrics, only the stair navigation trials of participants following a step-over-step pattern were considered. Strategy preference for the stair navigation tasks (step-by-step or step-over-step) for all groups and visits is also reported.

One-way ANOVAs with a Bonferroni correction were conducted to compare the patients’ performance to the controls’ (SPSS, IBM, USA). Repeated-measures ANOVAs were also used to assess differences between implant groups at each operative state. The significance level was not adjusted for comparisons and is reported at *P* < 0.05, *P*< 0.01 and *P* < 0.001. In reporting changes, an improvement (highlighted in green in the tables) was defined as a statistically significant change that led to a parameter’s average value being closer to the controls’ corresponding measurement, whilst the opposite was true for a deterioration in a metric (in red).

## Results

### Walking

With the exception of the post-operative performance of the UC group, all patients, both pre-operatively and post-operatively, had a lower walking speed (m/s) and cadence (steps/min) compared to the controls (Table 3, spatiotemporal parameters). Furthermore, both the CR DD and UCR groups walked with increased stride times during both patient visits. The only significant improvement in the spatiotemporal performance of the post-operative population was observed at the contralateral foot off instance (% of gait cycle) of the CR DD group (Table 3). Contralateral heel strike events (% of gait cycle) were consistent for all groups and visits, and all patient groups had significantly longer double support times compared to the control group.

**Table 3:**
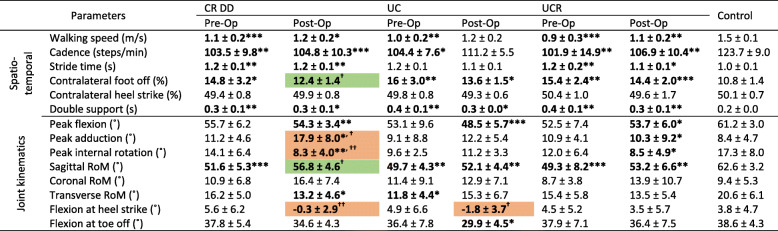
Knee spatiotemporal and kinematic parameters in walking

Flexion, adduction and internal rotation angles are indicated by positive values, while extension, abduction and external rotation by negative numbers

*/ ** / *** = significance at *p* <.05, <.01 and <.001 between implant and control groups, respectively

^**† / †† / †††**^ = significance at *p* <.05, <.01 and <.001 between pre-op and post-op visits

^***1,2,3***^ *= significance at p <.05, <.01 and <.001 between implant groups on the same visit*

**Fig. 3:**
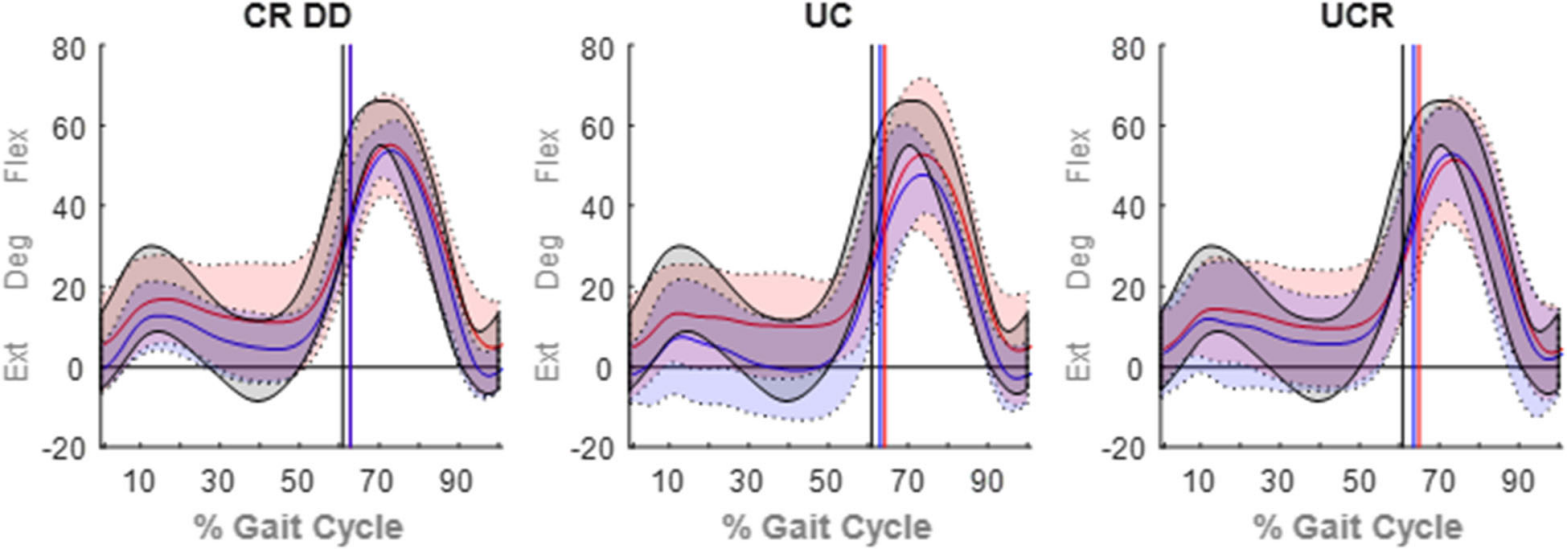
Average (solid lines) knee flexion ±2 standard deviations (shaded bands) for the pre-operative (in red), post-operative (in blue) and control (max/min in black; no average shown) groups. Toe off occurrences (% gait cycle) are indicated by vertical solid lines

Pre-operative peak knee joint angles of all implant groups were statistically comparable to the controls’ recordings (Table 3) but becoming statistically worse, post-operatively, compared to controls. The difference in knee flexion during the patients’ post-operative assessment, compared to before the TKA, was evident throughout the entirety of the gait cycle (Fig. 3). Furthermore, the post-operative relapse of the peak adduction and internal rotation angles (°) of the CR DD was statistically significant (Table 3). Generally, the patients’ coronal and transverse RoM (°) was unaffected by the TKA and comparable to the controls’ RoM. In contrast, only patients who received a CR DD implant showed a significant improvement in sagittal RoM (°) that was on a par with the control’s assessment (Table 3). Finally, both the CR DD and UC bearings displayed knee extension at heel strike, whilst the UC implant demonstrated a significantly reduced flexion at toe off, post-operatively (Table 3).

**Fig. 4:**
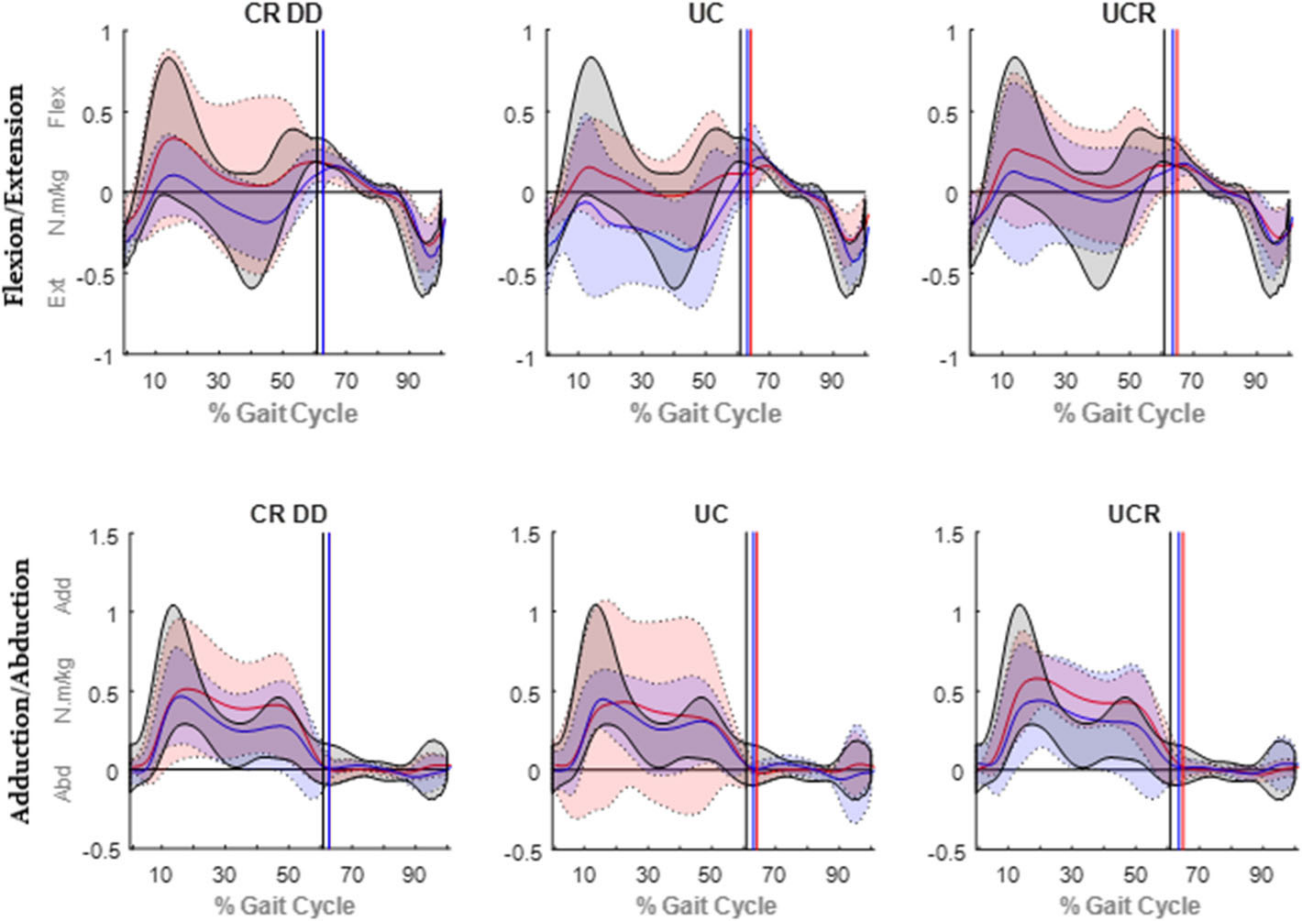
Average (solid lines) knee moments in walking and ±2 standard deviations (shaded bands) for the pre-operative (in red), post-operative (in blue) and control (black; no average shown) groups. Foot off occurrences (% gait cycle) are indicated by vertical solid lines

Knee flexion moment was typically biphasic in nature, with two flexion peaks at early and late stance, and an intermediate extension peak during mid stance (Fig. 4). Knee adduction/abduction moment also followed a similar pattern with two marginal adductions (Table 4) and one centralised abduction peak (Fig. 4). Throughout the entirety of the gait cycle, normalised pre-operative peak flexion/extension moments for all participants were similar; however, early stance moments for the CR DD group reduced after the operation (Table 4), whilst the analogous mid-stance extension moments increased for the CR DD and UC groups alike. On the whole, peak adduction/abduction moments, during all concerned phases of the gait cycle and patient visits (Table 4), were comparable to the control values, an exemption being the recorded moments in mid-stance from the CR DD group that were significantly increased post-operatively. Finally, both normalised peak power estimates were significantly reduced compared to controls, for all implant types and visits, except for the peak eccentric power of the UCR group at both visits (Table 4).

**Table 4:**
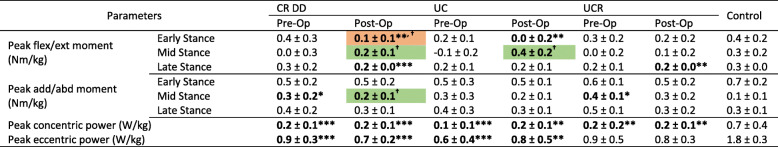
Knee kinetic parameters in walking

*/ ** / *** = significance at *p* <.05, <.01 and <.001 between implant and control groups, respectively

^**† / †† / †††**^ = significance at *p* <.05, <.01 and <.001 between pre-op and post-op visits

### Stair navigation

As opposed to the post-operative measurement, pre-operative walking speed in stair navigation was consistently lower compared to the controls’ (Tables 5 and 6**)**. Similarly, pre-operative significant differences in the cadence and stride time of the osteoarthritic patients disappeared post-operatively. The CR DD group demonstrated a delayed contralateral foot off timing during stair descent, significantly different to all other study groups (Table 6) which also persisted post-operatively. As for the contralateral heel strike, the pre-operative performance of the UCR group during stair ascent was also significantly different 1 year after surgery (Table 5). For the UC group during stair ascent and descent, and the CR DD group in descent, the pre-operative double support phase was significantly longer compared to the control group, yet the UC implant group exhibited a post-operative reduction in double support time (Table 6).

**Table 5:**
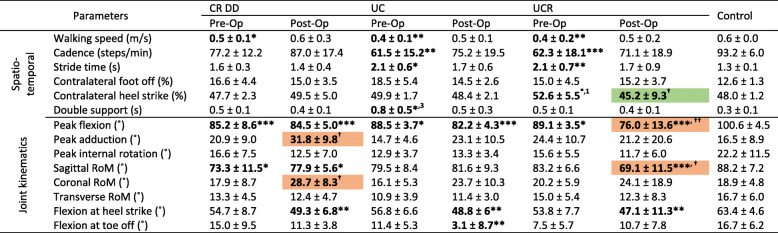
Knee spatiotemporal and kinematic parameters in stair ascent

Flexion, adduction and internal rotation angles are indicated by positive values, while extension, abduction and external rotation by negative numbers

*/ ** / *** = significance at p <.05, <.01 and <.001 between implant and control groups, respectively

^**† / †† / †††**^ = significance at p <.05, <.01 and <.001 between pre-op and post-op visits

^**1,2,3**^ = significance at p <.05, <.01 and <.001 between implant groups on the same visit.

**Table 6:**
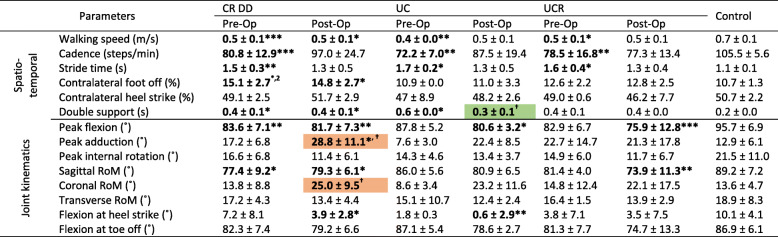
Knee spatiotemporal and kinematic parameters in stair descent

Flexion, adduction and internal rotation angles are indicated by positive values, while extension, abduction and external rotation by negative numbers

*/ ** / *** = significance at *p* <.05, <.01 and <.001 between implant and control groups, respectively

^**† / †† / †††**^ = significance at *p* <.05, <.01 and <.001 between pre-op and post-op visits

^**1,2,3**^ = significance at *p* <.05, <.01 and <.001 between implant groups on the same visit

The control group demonstrated greater peak flexion angles (°) during stair ascent (Table 5) than all patient groups in all instances. Furthermore, the peak flexion angle of the patients with a UCR bearing decreased 1 year after surgery. Even though the pre-operative peak flexion angles of the UC and UCR groups were equivalent to the control group in stair descent (Table 6), their function declined post-operatively. The adduction angles of the CR DD group significantly increased post-operatively for both stair ascent and descent (Tables 5 and 6), whilst there were no evident differences in peak knee internal rotation angles between groups.

Knee sagittal RoM was higher in stair navigation than during walking. For both stair tasks, the sagittal RoM of the control group was greater than the patients, but only the UCR implant group exhibited a significant post-operative loss of sagittal RoM during stair ascent (Table 5). The CR DD group showed a large and statistically significant post-operative increase in the frontal RoM in both ascent and descent (Tables 5 and 6). Finally, except for the UCR group during stair descent, all TKA designs resulted in a straighter leg at heel strike post-operatively compared to the control group.

With regard to the type of movement strategies that were adopted to complete the stair navigation assessment, all control participants paced with a step-over-step strategy, whilst 22% and 38% of the pre-operative recordings showed patients walking step-by-step during stair ascent and descent, respectively (Table 7). TKA surgery also shifted the patients’ movement preferences towards the behaviour of the controls during stair ascent (Pearson Chi-square, *P*=0.016) but not during descent (*P*=0.282), in 3% and 12% of the post-operative trials was the step-by-step strategy used whilst ascending and descending, respectively.

**Table 7:** Step-by-step strategy preference for all groups and visits

	CR DD		UC		UCR		All patients		Control
	Pre-Op	Post-Op	Pre-Op	Post-Op	Pre-Op	Post-Op	Pre-Op	Post-Op	
Stair ascent	8%	0%	24%	0%	43%	9%	22%	3%	0%
Stair descent	17%	12%	60%	0%	57%	21%	38%	12%	0%

## Discussion

This study compared the functional performance of healthy controls and patients who received one of three randomly assigned B. Braun Columbus® knee implants: a low congruency fixed (CR DD), a high congruency fixed (UC), or a high congruency mobile (UCR) bearing. This work is the first in the literature to assess the UCR Colombus mobile bearing in vivo and compare the performance of different Colombus knee prostheses in a series of activities of daily living using motion capture. Spatiotemporal, kinematic and kinetic parameters were compared between all patient groups and controls, between pre-operative and post-operative assessments (intra-implant changes), and between implant groups of the same operative state (inter-implant changes). Participant demographics indicated that controls were similar in age to all patient groups but with a significantly lower BMI (Table 2).

### Differences between patient and control groups

Statistically significant differences were primarily observed between patient and control groups, rather than amongst implant designs or patient visits. On the whole, patients walked at a slower speed and cadence, and with greater periods of double support. Such differences suggest less confidence in the lower limbs and a reluctance to remain in single support, possibly due to joint pain and discomfort. Although the magnitude of these inequalities was lessened after surgery and the OKS were improved post-operatively, patients’ mobility and performance 1 year after surgery had not yet reached the controls’ level.

The controls’ gait generally featured greater peak knee flexion and rotation angles and lower adduction angles compared to the patient movements across all activities and visits (Tables 3, 5, and 6). Accordingly, controls consistently demonstrated greater sagittal and transverse RoM during all recordings, whilst the coronal RoM was lower than the patient participants in most comparisons. As for the knee flexion at heel strike and toe off, control volunteers generally exhibited higher flexion angles, particularly compared to the patients’ post-operative performance. Low knee flexion angles, predominantly during heel strike events in stair navigation, may indicate loss of function and lack of confidence in the operated joint, since straight rather than flexed knee joints may offer more stability in gait initiation. Further, increased knee flexion angles at toe off indicate greater ground clearance prior to swing, thus diminishing the likelihood of tripping and falling.

Knee flexion/extension moments for level walking showed no variability between controls and pre-operative patient participants. Peak adduction moments in early and late stance were also similar across all groups and visits. In contrast, pre-operative peak abduction moments during mid stance were higher for patients compared to the controls; generally, these peak moments were reduced after surgery, whilst the transition of the CR DD group was also statistically significant (Table 4). With the sole exception of the UCR group, peak power magnitudes in walking were significantly lower during all patient visits as compared to the controls.

### Intra-implant changes

In general, patients’ functional performance improved following TKA: there were thirty-five statistically significant differences in the spatiotemporal parameters between pre-operative patients and controls, in contrast to fourteen post-operatively. However, measurements such as walking speed and double support duration never attained statistical equivalence with the control group, suggesting that mobility impairments persisted after surgery. Yet, it is worth mentioning that functional improvements can be made beyond a year after TKA. On three occasions, spatiotemporal parameters were significantly improved 1 year after surgery: the point of contralateral foot off during walking was significantly earlier for the CR DD group, and so was the contralateral heel strike of the UCR group in stair ascent; finally, the period of double support was significantly shorter for the UC implant group in stair descent (Tables 3, 5, and 6, in green). Since both the contralateral heel strike and foot off determine the period of double support, these advancements in the performance of all implant groups imply a degree of confidence in the operated limb to remain longer in single stance support.

In terms of joint kinematics, post-operative patient recordings displayed a generic increase in knee sagittal and coronal RoM, yet the post-operative peak knee flexion and rotation angles were broadly reduced from their pre-operative countervalues, implying that the post-operative improved sagittal RoM is due to higher knee extension rather than flexion. Moreover, abnormally high knee adduction angles and coronal RoM (e.g., Tables 5 and 6) are not entirely unexpected: in a pre-operative significantly varus knee, the lateral collateral ligaments may have less strain when loaded in comparison to when the knee is restored to neutral, and the collateral ligaments may be more lax postoperatively. Post-surgery patients displayed a straighter leg at heel strike and toe off, whilst in two instances (CR DD and UC group in walking) the post-operative loss of function for these two parameters was statistically significant.

The post-operative reduced knee flexion angles during heel strike and toe off may have shifted the knee’s flexion axis closer to vertical component of the ground reaction force, thus reducing the knee’s flexion/extension moment during early and late stance. This was evident in the patients’ peak flexion moments in early stance that were generally reduced post-operatively (Table 4), possibly due to the reduced flexion angles at heel strike (Table 3). The UCR group did not show any significant shift in its performance after surgery, whilst in contrast the other two patient groups exhibited changes in the moment peaks during early and mid-stance (Table 4). Peak adduction/abduction moments were largely unaffected by the TKA, and only the CR DD witnessed an improvement in the magnitude of the peak abduction moments in mid stance. Finally, patients’ post-operative peak power values were in all cases comparable to their pre-operative assessment but consistently lower than the control group.

All healthy controls adopted the step-over-step stair navigation strategy, whilst fewer patients preferred the step-by-step movement strategy after TKA surgery while ascending (Table 7, *P*=0.282), signifying that their post-operative functional improvement was also reflected in their movement behaviour. TKA and osteoarthritis patients’ movement behaviour were also previously assessed for other activities of daily living by the same authors [21, 22], similarly indicating that patient participants favour different movement strategies compared to asymptomatic controls.

### Inter-implant changes

Statistically significant differences between implant design groups were only observed pre-operatively (Tables 5 and 6): the contralateral heel strike in stair ascent for the UCR group, the double support duration in stair ascent for the UC group, and the contralateral foot off in stair descent for the CR DD group. None of these differences persisted 1 year after surgery.

When comparing the total number of parameters that significantly differed between pre-operative and post-operative visits (Tables 3, 4, 5 and 6, key: †), the CR DD implant showed an improvement in four parameters (green-shaded values) and a deterioration in eight (red-shaded values). The UC group had two improved metrics (peak flexion/extension moment in mid stance during walking and double support time during stair descent) and one impaired (flexion at heel strike during walking). Whilst patients with a UCR bearing showed a functional improvement in one parameter (contralateral heel strike % during stair ascent), they also had an adverse change in two (peak flexion and sagittal RoM in stair ascent). In view of the above gross metrics, the UC implant showed an overall functional improvement, whilst the CR DD and UCR bearings were accompanied with an overall deterioration in the patients’ function.

The sagittal RoM of the low congruent-fixed knee bearing (CR DD) showed a statistically significant post-operative increase (Table 3, walking trials). Additionally, the same implant group exhibited significantly greater post-operative coronal RoM in both stair ascent and descent, to an extent that it was regarded as a functional disadvantage (Tables 5 and 6, in red). This was anticipated since, in low congruency bearings, the sagittal femoral radius is decreased in the high end of the flexion range, thus improving the knee’s RoM. By contrast, patients with UCR implants did not exhibit an improved tibiofemoral axial rotation during either activity of daily living, despite the design’s enhanced transverse rotational freedom of the polyethylene insert on the tibial plateau. Finally, the RoM of the high congruent fixed bearing (UC) on all axes was neither significantly increased nor decreased, for all recorded activities.

### Study limitations

A limitation of this study emerges from the sample size of the participating population which was further reduced by the high percentage of withdrawals and no-shows, also leading to uneven sample sizes between groups. Even if the study is likely underpowered, a sample size of 24 patients is reasonable for a motion capture study that follows TKA patients for a year and is comparable to previous research on this topic [18, 23, 24]. Additionally, the significant difference in the BMI of the patient and control participants may have significantly influenced the kinematic and kinetic recordings in this work (as described by [25]). Finally, in order to gauge the functional performance of the investigated implant designs, a number of spatiotemporal, kinematic, and kinetic parameters were employed. Even though all studied parameters are relevant to human gait and may be used to distinct natural from pathological movements, they do not carry equal weight in the evaluation of an implant’s post-operative functional performance. Additionally, several statistically significant changes amongst patient groups may be regarded as negligible during activities of daily living (e.g., Table 5, increased contralateral heel strike timing of the UCR group) and are potentially clinically insignificant. Therefore, the interpretation of this study’s findings is also subject to the clinical significance of each metric.

## Conclusion

Generally, TKA surgery largely improved the function of all patient groups. Nevertheless, metrics such as the walking speed, double support time and peak power magnitudes never became statistically equivalent to the controls’ performance. In terms of the net number of positive post-operative changes, the UC implant provided better post-operative function compared to the other two implants in this study, followed by the UCR and CR DD bearings. Nevertheless, the CR DD group showed the most prominent post-operative improvement during the walking assessment in the most commonly reported functional metric, the sagittal RoM. The enhanced operation range of the CR DD design was attributed in its mechanical properties as a low congruent bearing. Finally, this study is the first in the literature to assess the Columbus UCR ultra-congruent system with a rotating platform. It was hypothesised that the UCR design would permit more natural movements compared to the other two implants when carrying out everyday activities. Yet, both the UC and UCR groups showed comparable functional performance and improvement after TKA surgery and a similarly restricted post-operative sagittal RoM. Patients with a UCR implant did not exhibit an improvement in their tibiofemoral axial rotation despite the bearing’s mobile design.

## Supplementary Information

**Additional file 1:.** Table 1 Knee kinetic parameters in stair ascent.

**Additional file 2:.** Table 2 Knee kinetic parameters in stair descent.

## Data Availability

Not applicable.
