# Supplementary material for: Implant design affects walking and stair navigation after total knee arthroplasty: a double-blinded randomised controlled trial
Source: J Orthop Surg Res. 2021 Mar 6;16:177. doi: 10.1186/s13018-021-02311-x (PMC7936496; doi:10.1186/s13018-021-02311-x)
Supplement: Supplementary file 2 — Additional file 2:. Table 2 Knee kinetic parameters in stair descent. [file 13018_2021_2311_MOESM2_ESM.docx]

**Table 2** Knee kinetic parameters in stair descent.

| Parameters | | CR DD | | UC | | UCR | | Control |
| --- | --- | --- | --- | --- | --- | --- | --- | --- |
|  |  | Pre-Op | Post-Op | Pre-Op | Post-Op | Pre-Op | Post-Op |  |
| Peak flex/ext moment (Nm/kg) | Early Stance | 0.3 ± 0.2 | 0.1 ± 0.1 | 0.1 ± 0.0 | 0.0 ± 0.1 | 0.4 ± 0.1 | 0.4 ± 0.1 | 0.6 ± 0.3 |
|  | Mid Stance | -0.1 ± 0.2 | 0.1 ± 0.3 | 0.0 ± 0.0 | 0.1 ± 0.1 | -0.2 ± 0.2 | -0.3 ± 0.0 | -0.2 ± 0.2 |
|  | Late Stance | 0.5 ± 0.2 | 0.4 ± 0.1 | 0.4 ± 0.0 | 0.2 ± 0.0 | 0.5 ± 0.1 | 0.6 ± 0.1 | 0.7 ± 0.3 |
| Peak add/abd moment (Nm/kg) | Early Stance | 0.5 ± 0.3 | 0.3 ± 0.1 | 0.3 ± 0.0 | 0.4 ± 0.1 | 0.6 ± 0.1 | 0.4 ± 0.1 | 0.6 ± 0.3 |
|  | Mid Stance | 0.2 ± 0.1 | 0.2 ± 0.1 | 0.1 ± 0.0 | 0.1 ± 0.1 | 0.3 ± 0.1 | 0.2 ± 0.0 | 0.2 ± 0.1 |
|  | Late Stance | 0.3 ± 0.1 | 0.3 ± 0.1 | 0.3 ± 0.0 | 0.3 ± 0.1 | 0.4 ± 0.0 | 0.4 ± 0.1 | 0.5 ± 0.2 |
| Peak concentric power (W/kg) | | **0.2 ± 0.1*** | 0.2 ± 0.1 | 0.2 ± 0.0 | 0.3 ± 0.4 | 0.3 ± 0.0 | 0.2 ± 0.1 | 0.7 ± 0.4 |
| Peak eccentric power (W/kg) | | 0.4 ± 0.3 | 0.5 ± 0.3 | 0.5 ± 0.0 | 0.7 ± 0.5 | 0.4 ± 0.2 | 0.5 ± 0.2 | 0.5 ± 0.2 |
| Flexion, adduction and internal rotation angles are indicated by positive values, while extension, abduction and external rotation by negative numbers.  */ ** / *** = significance at p <.05, <.01 and <.001 between implant and control groups, respectively. | | | | | | | | |
